# Laser-Assisted Production of Carbon-Encapsulated Pt-Co Alloy Nanoparticles for Preferential Oxidation of Carbon Monoxide

**DOI:** 10.3389/fchem.2018.00487

**Published:** 2018-10-16

**Authors:** Gema Martinez, Ana Malumbres, Angela Lopez, Reyes Mallada, Jose L. Hueso, Jesus Santamaria

**Affiliations:** ^1^Networking Research Center on Bioengineering, Biomaterials and Nanomedicine, CIBER-BBN, Zaragoza, Spain; ^2^Department of Chemical Engineering, Nanoscience Institute of Aragon (INA), Universidad de Zaragoza, Zaragoza, Spain

**Keywords:** bimetallic alloy, nanoparticles, Pt_x_Co_y_/C catalyst, laser pyrolysis, preferential CO oxidation

## Abstract

C-encapsulated highly pure Pt_x_Co_y_ alloy nanoparticles have been synthesized by an innovative one-step *in-situ* laser pyrolysis. The obtained X-ray diffraction pattern and transmission electron microscopy images correspond to Pt_x_Co_y_ alloy nanoparticles with average diameters of 2.4 nm and well-established crystalline structure. The synthesized Pt_x_Co_y_/C catalyst containing 1.5 wt% of Pt_x_Co_y_ nanoparticles can achieve complete CO conversion in the temperature range 125–175°C working at weight hourly space velocities (WHSV) of 30 L h^−1^g^−1^. This study shows the first example of bimetallic nanoalloys synthesized by laser pyrolysis and paves the way for a wide variety of potential applications and metal combinations.

## Introduction

Increasing interest is currently being devoted to the use of supported bimetallic alloy and intermetallic nanoparticles as a promising way to modify activity and selectivity, to improve stability and partially substitute expensive noble metals in conventional supported metallic catalysts, such as Pt, Pd, and Rh system (Yu et al., 2012; Furukawa and Komatsu, 2017). In general, the properties of the bimetallic catalysts differ from their monometallic counterparts owing to the synergistic effects of geometry and electronic effects between metals. Therefore, their catalytic performance can be tuned, because an additional degree of freedom, for modifying the geometric and electronic structures is applicable by changing their composition and size (Tao et al., 2012; Wang et al., 2014).

Pt_x_Co_y_ alloy is one of the most widely studied bimetallic systems because of its stability and broad catalytic scope (Service, 2002; Stamenkovic et al., 2007; Huang et al., 2017). One of the reactions in which these multimetallic alloys may offer greater benefits than conventional Pt catalysts is the preferential oxidation (PROX) of carbon monoxide (CO; Liu et al., 2012), a case of substrate-selective oxidation of CO in excess of hydrogen (H_2_). The main requirements to be fulfilled by the catalysts used in PROX reaction are: (i) high activity at low temperatures achieving CO conversion to CO_2_ higher than 99%, (ii) high selectivity to the CO oxidation reaction, avoiding the oxidation of hydrogen in a wide operation temperature window (e.g., 80–180°C) between the low temperature shift reactor operation around 200°C and (iii) the low temperature feed to the PEMFC. Hence the catalyst should not be deactivated by the presence of CO_2_ and H_2_O in the reformate feed stream (Liu et al., 2012). In fact, an improved performance in the catalytic activity for this reaction when using Pt_x_Co_y_ alloy catalysts, instead of Pt/C, is observed as a consequence of the special synergic effect between cobalt and platinum (Yan et al., 2004; Ko et al., 2007; Snytnikov et al., 2007; Wang et al., 2013; Furukawa et al., 2016). The formation of the intermetallic alloy induced an extension of the Pt-Pt atomic distance and electron transfer from Pt to Co. Both effects result in weaker adsorption of CO, compared to the pure metal resulting in higher activity at lower temperature (Komatsu and Tamura, 2008). However, there are still serious limitations in their synthesis, because of the difference in standard reduction potential between the two metals ions and the distinct atom sizes. A challenge is to maintain simultaneously (i) a narrow nanoscale size distribution; (ii) a uniform composition throughout the nanoparticles; (iii) a fully alloyed degree, and (iv) high dispersion on the support, which represents a serious challenge in conventional synthesis approaches.

Routine synthesis approaches typically involve the impregnation of an already synthesized carbon-supported Pt metal catalyst with a second metal precursor salt, followed by alloying at high temperatures (≥900°C) under inert gas or reducing conditions (Tamizhmani and Capuano, 1994; Min et al., 2000; Takenaka et al., 2010). Nevertheless, this thermal treatment gives rise to undesired particle growth by sintering and coalescence of the particles with the concurrent increase of average particle size, resulting in a lower catalysts active area (Antolini, 2003). As an alternative to overcome these problems, other procedures such as reduction of Pt and the second metal at low temperature (Xiong et al., 2002), micro emulsion methods (Xiong and Manthiram, 2005) or polyol methods (Jang et al., 2011) are aimed to synthesize bimetallic nanoparticles under milder conditions. However, these methods offer low alloying degree (Zignani et al., 2008; Jang et al., 2011; Vinayan et al., 2012; Lopez et al., 2016), mainly because the formation of alloyed intermetallic phases generally needs high temperatures(Furukawa and Komatsu, 2017). In addition, these processes require longer synthesis time (including cleaning protocols) and deployment on supports.

Alternatively, multimetallic nanoparticles have been also produced in gas phase by procedures such as flame pyrolysis (Strobel et al., 2005), laser ablation (Senkan et al., 2006) or plasma-assisted dissociation of organometallic vapor (Lin and Sankaran, 2011; Saedy et al., 2017). Unfortunately, these gas-phase approaches have also suffered from excessive particle growth and aggregation, reducing the surface area and number of actives sites (Rodriguez et al., 2011; Wang and Li, 2011). Therefore, in order to preserve the catalytic properties of the nanoparticles, particle agglomeration must be prevented. For this purpose, encapsulating shells appear as a promising and elegant strategy. Among these encapsulating candidates, carbon offers many advantages, such as high stability under various physical and chemical conditions, high electrical conduction for electrochemical applications, and low manufacturing cost. Various techniques for carbon encapsulation of metal nanoparticles have been investigated including laser ablation of organometallic targets (Munoz et al., 2010; Seral-Ascaso et al., 2013), solution plasma processes (Kang et al., 2013), one-step pyrolysis using cyanamide and metal salts as precursor (Han et al., 2015) or one-pot light-assisted evaporation induced self-assembly approach (Ghimbeu et al., 2015). Unfortunately, most of them render broad particle size distribution or at least less controllable and limited availability of control in stoichiometry requirements, making them very restricted methods for the synthesis of alloy nanoparticles.

Therefore, further developments are still needed in order to design a versatile one-pot synthesis that allows high nanoparticle dispersion, tuneable stoichiometry and at the same time preserving small particle size and achieving good alloying and stability. In this work, we propose a simple one-pot synthesis method for alloying nanoparticles containing C shells, based on laser pyrolysis processing. Previous studies in our group have shown that this technique could be used to successfully synthesize a variety of nanoparticles with extremely high purity (Martínez et al., 2012; Malumbres et al., 2015). Although most of the research in this area is limited to metal precursors which are gases or liquid having sufficient vapor pressure at moderate temperature, which restricts its application to certain elements or makes necessary to synthesize specific organometallic precursors or even use highly flammable and toxic precursors as it is the case of silane for the synthesis of Si nanoparticles. Recently, much attention is being focused on the interaction between the laser beam and the aerosols droplets containing solid precursors, that may expand the application of the technique to all the elements as far as their salts are soluble in spray-able solvents (Wang et al., 2017). The most important merits of laser pyrolysis processing include the well-defined interaction volume, spatial uniformity of the reaction zone, short millisecond scale residence times, high heating/cooling rates, the fact that the nanoparticles properties can be tuned by adjusting the process parameters, the continuous nature that avoids the intrinsic variability of batch processing and the high purity of the prepared materials.

In this paper we present a flexible and continuous Pt_x_Co_y_/C catalysts synthesis, which is much more time and cost saving than current multi-step processes. As far as we are concerned this is the first time that bimetallic nanoparticles have been synthesized by laser pyrolysis. The innovative, versatile, and continuous single step strategy involves interaction between the laser beam and the organometallics precursors in a liquid spray form. The role of the solvent it is not only to transport the solid metal precursors at the reaction zone but also to provide the source to form the carbon framework avoiding agglomerating of the particles. By carefully combining precursor's ratio, bimetallic nanoparticles of controlled composition can be tailored by this approach. The chemical and structural characterization of the synthesized material revealed that the obtained Pt_x_Co_y_ alloy nanoparticles were encapsulated in C matrix and exhibited a uniform size distribution, average diameters below 3 nm, and high crystallinity. Finally, the Pt_x_Co_y_/C (Pt:Co, 3:1) nanoparticles were deposited on a ETS-10 microporous support (1.5 wt.% Pt_x_Co_y_/C) and their catalytic activity was tested in PROX reaction feeding a simulated steam reforming stream (1%CO, 21%CO_2_, 3%H_2_O, 1%O_2_, and H_2_ balance).

## Materials and methods

### Chemicals

Platinum (II) acetylacetonate [Pt(acac)_2_, 97%], cobalt (III) acetylacetonate [Co(acac)_3_, 99.99%], toluene (99.5%) and absolute ethanol (≥98%) were supplied from Sigma Aldrich and used without further purification.

### Pt_x_Co_y_/C composite nanoparticles synthesis

**Scheme 1:**
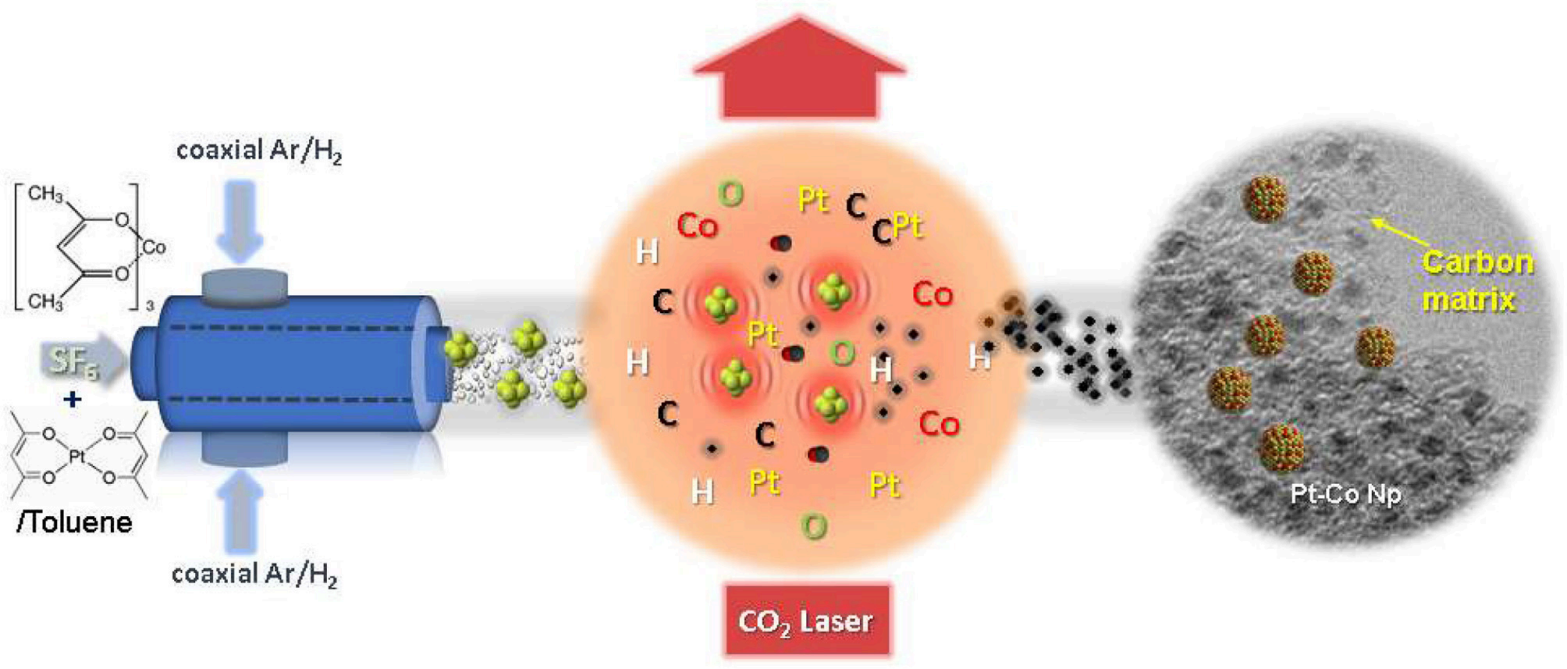
Schematic diagram for the synthesis of carbon-encapsulated Pt_x_Co_y_/C bimetallic nanoparticles by laser pyrolysis.

The synthesis of Pt_x_Co_y_/C composite nanoparticles with different compositions has been carried out by laser pyrolysis in a continuous flow reactor described elsewhere (Martínez et al., 2012; Malumbres et al., 2013, 2015). The starting solution employed for the synthesis of Pt_x_Co_y_/C was prepared by dissolving Pt(acac)_2_ (47 mg, 0.12 mmol) and the corresponding amount of Co(acac)_3_ [11 mg (0.04 mmol) or 30 mg (0.12 mmol)] in toluene, to get a molar ratio Pt:Co = 3 or Pt:Co = 1. The technique is based on the interaction between the laser beam (Rofin SCx30, λ = 10.6 μm) at 100 W power and the starting materials in a liquid spray form (aerosol). Sulfur hexafluoride (SF_6_) was added as sensitizer gas. The liquid mixture was introduced in the reactor by a syringe pump working at 15 ml/h. The aerosol spray was produced by a nebulizer immediately located before the chamber. Aerosol droplets were transported in a flow of Ar/SF_6_, 130 and 30 sccm, respectively, into the reaction chamber through a ¼ inch inner diameter nozzle. A flow of hydrogen (10 sccm) and argon (100 sccm) was used as coaxial gas flow to confine the reaction in a very small volume without any interaction with reactor walls. The aerosol and laser beam are designed to intersect orthogonally, and the beam diameter (d_laser_) and the aerosol spray diameter (d_spray_) were controlled in such way that d_spray_ < d_laser_. This configuration ensured that all the spray molecules were confined within the beam area to complete the pyrolysis (see Scheme 1). In order to prevent powder deposition onto the vertical and horizontal windows they were continuously flushed with 600 and 200 sccm of Ar and N_2_, respectively. The pressure was maintained constant at 200 mbar through a diaphragm valve located between the reaction chamber and the vacuum pump. The gas flows were controlled by mass flow controllers. The reaction proceeded with color change from green light (color of the precursor solution) to black (color of the nanostructures powder obtained). To capture and isolate nanoparticles, the freshly nucleated particles were directly collected onto cellulose filters (F2044). Typical values and ranges of the experimental parameters are listed in Table 1. This work has been performed by the ICTS “NANBIOSIS” by the Synthesis of Nanoparticles Unit of the CIBER in Bioengineering, Biomaterials &Nanomedicine (CIBER-BBN) at the Institute of Nanoscience of Aragon (INA)-Universidad de Zaragoza.

**Table 1:** Experimental parameters for the optimized synthesis of carbon-encapsulated Pt_x_Co_y_ bimetallic nanoparticles.

**Synthesis conditions**	
Flow rate of windows gases (sccm)	800
Flow rate of coaxial gas Ar/H_2_ (sccm)	100/10
Aerosol spray flow Ar/SF_6_ (sccm)	130/30
Precursor solution flow (mL/h)	15
Power of laser beam (W)	100
Working pressure (mbar)	200

### Structural characterization

A battery of techniques was used to characterize the Pt_x_Co_y_/C composite nanoparticles. Particle morphology and size distribution were determined by a FEI Tecnai thermoionic transmission electron microscopy (TEM) operated at 200 kV. More than 200 particles were measured to evaluate the mean diameter. The data were fitted with a normal distribution function and the standard deviation was obtained for all the samples. To determine the crystalline structure and the composition of the particles, High Resolution Transmission Electron Microscope (HRTEM), Scanning Transmission Electron Microscope with high angle annular dark field (STEM-HAADF) and Energy Dispersive X-ray Spectroscopy (EDS) analysis were performed by using a FEI TECNAI F30 and FEI Titan™ Cube (80–300 kV) microscopes at an acceleration voltage of 300 kV. To prepare the sample, 10 μL of a ethanol particle suspension were dropcasted on a lacey carbon TEM grid. Powder X-ray diffraction (XRD) analyses were performed in a Rigaku/Max System diffractometer with Cu Kα radiation source (λ = 0.15418 nm). Surface composition was analyzed by X-ray photoelectron spectroscopy (XPS) with an Axis Ultra DLD (Kratos Tech.) The spectra were excited by a monochromatic Al Kα source (1486.6 eV) run at 12 kV and 10 mA and pass energy of 20 eV was used. The binding energies were referenced to the internal C 1 s (284.6 eV) standard. Analysis of the peaks was performed with CasaXPS software, using a weighted sum of Lorentzian and Gaussian component curves after Shirley background subtraction. To characterize the carbon shell of the nanoparticles, Raman spectra were obtained with a Laser Raman WiTec Alpha 300 spectrometer, with the 532 nm line of an Ar^+^ ion laser. The bulk chemical composition of digested samples was analyzed using microwave plasma atomic emission spectroscopy (Agilent 4100 MP-AES).

### Catalytic activity tests

The as synthesized Pt_x_Co_y_ nanoparticles were incorporated to the microporous titanosilicates ETS-10 support by incipient wetness impregnation method, to get 1.5 wt.% Pt_x_Co_y_, following our previous protocol (Lopez et al., 2016). The activity measurements were carried out in an experimental set-up described elsewhere (Lopez et al., 2016). The supported catalyst, 100 mg together with 200 mg of quartz, used as diluent, was loaded in a quartz tube of 9 mm internal diameter. The reaction temperature was measured with a thermocouple located in the center of the catalyst bed. The composition of the feed stream was: 1% CO, 1% O_2_, 3% H_2_O, 21% CO_2_, and hydrogen balance. The feed flow was 50 STP mL/min which corresponds to a (weight hourly space velocity) WHSV = 30 L h^−1^g^−1^. Prior to catalytic activity tests, the solids were heated in air at a heating rate of 5°C/min, up to 500°C and kept at this temperature for 3 h. Then the catalyst was cooled down to room temperature under N_2_ atmosphere. Feed and products were analyzed by gas chromatography with a Varian CP-4,900 Micro-GC equipped with two modules containing, molecular sieve and Pora PLOT Q columns, respectively and using helium as carrier gas. Under the analysis conditions the detection limit of CO was 5 ppm.

The CO conversion and the selectivity for the PROX reaction were calculated according to:

$$\begin{array}{lll} X_{co} & = & \frac{F_{CO\;feed}-F_{CO\;outlet}}{F_{CO\;outlet}} \end{array}$$

$$\begin{array}{lll} S_{co_{2}} & = & \frac{2\left(F_{CO\;feed}-F_{CO\;outlet}\right)}{\left(F_{O_{2}feed}-F_{O_{2}outlet}\right)} \end{array}$$

Being *F*_*i*_ the corresponding molar flow of each component. The conversion values were obtained after the reactor temperature was stable for at least 30 min. and the reported values represent the average of 3 samples taken at the reactor exit. CH_4_ was not detected in any of the experiments carried out. The C mass balance was measured and was always in the range 98–102%.

## Results and discussion

### Synthesis and characterization of Pt-Co nanoalloys

As a first step toward accessing carbon dispersed bimetallic alloy nanoparticles, we separately studied the conversion of individual acetylacetonate metal precursors [Pt(acac)_2_ and Co(acac)_3_] into the corresponding monometallic nanoparticles. Figure S1 indicates that both organometallics precursors were successfully decomposed under similar experimental conditions (Table 1 in experimental section) to yield highly uniform and well-dispersed monometallic nanoparticles within a carbonaceus matrix. The nanoparticles are characterized by spherical shapes, narrow size distributions and particles diameters from ~2.5 to 3.5 nm.

Once the experimental conditions, for the pyrolysis of the aerosol, were established, the synthesis of Pt_x_Co_y_/C composite nanoparticles was accomplished by one-step pyrolysis of a toluene solution containing a mixture of both Pt(acac)_2_ and Co(acac)_3_ precursors in the required stoichiometric amount. As illustrated in Scheme 1, the fabrication process involves interaction between the laser beam and the starting materials in a toluene spray. When the laser beam intersects the SF_6_/Ar nebulized toluene solution reactant stream, a fast-atomic decomposition of the organometallic precursors is produced into the reaction area resulting in the formation of well-dispersed Pt_x_Co_y_ bimetallic nanoparticles within a carbonaceous shell.

**Figure 1:**
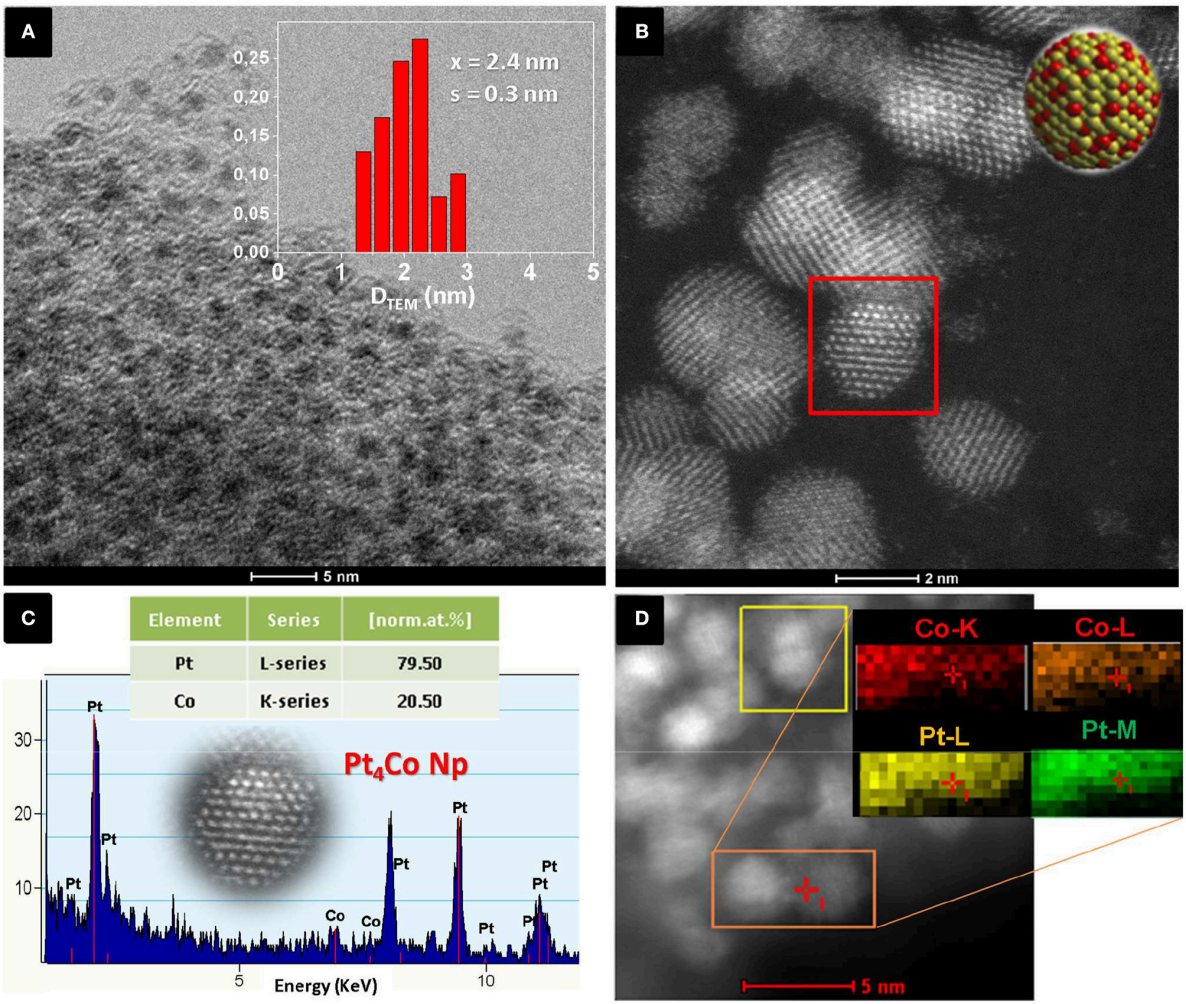
**(A)** Representative TEM image of Pt_4_Co nanoparticles encased in a C matrix, (*inset*) Particle size histogram, **(B)** STEM-HAADF image of the Pt_4_Co/C nanoparticles synthesized when the molar ratio of starting Pt:Co precursors is set to 3, **(C)** EDX analysis of an individual particle (selected area in **B**) indicating an experimental atomic ratio of Pt to Co of ~4 (Pt_4_Co/C), and **(D)** EDS mapping of elemental Pt (green) and Co (red) with corresponding STEM-HAADF image of the Pt_4_Co/C composite nanoparticles.

Figure 1A shows the TEM image and particle size distribution (inset Figure 1A) of the Pt_x_Coy/C obtained nanoparticles when the molar ratio of Pt to Co precursors was 3. The TEM image (Figure 1A) reveals that numerous individual nanoparticles with mean size of 2.4 ± 0.3 nm (inset) are well dispersed and perfectly C-encapsulated. Figure 1B shows a STEM-HAADF image (the contrast depends directly on the atomic number Z^2^), where the bright bimetallic nanoparticles are easily spotted against the C matrix. The nanocrystals appear as well-crystallized spherical particles. The semi-quantitatively chemical composition of the nanoparticles was determined by energy-dispersive X-ray (EDX) spectroscopy (Figure 1C). The analysis of the elemental distribution within a single nanoparticle (selected area on Figure 1B) corroborates the coexistence of both Pt and Co with atomic percentages of 79.5 ± 0.7 and 20.5 ± 0.5%, respectively. The EDS also shows high resolution 2D mapping of elemental Pt and Co (Figure 1D), which are homogeneously distributed throughout the entire nanoparticle. The bulk composition of the sample determined by Inductively coupled plasma (ICP) further confirmed an overall atomic ratio Pt:Co of 3.97 ± 0.04. The experimental ratios (Pt:Co = 4) determined by both measurements matched very well and were slightly higher than expected from the initial molar ratio fed to the reactor (Pt:Co = 3). The Pt-enrichment of the as-prepared Pt_4_Co/C composite nanoparticles can be tentatively attributed to different physicochemical features, such as different decomposition temperature of the organometallic acetylacetonates (Vonhoene et al., 1958). On the other hand, carbon is segregated and deposits as a mixture of amorphous carbon with randomly distributed graphitic domains, confirmed by Raman spectroscopy (Figure S2). The peak centered at 1.595 cm^−1^ (G-band) is assigned to graphite (Ferrari, 2007), while the peak at 1.348 cm^−1^ is the D-band mainly derived from the disordered carbon structures (Arbizzani et al., 2011; Liu et al., 2015).

**Figure 2:**
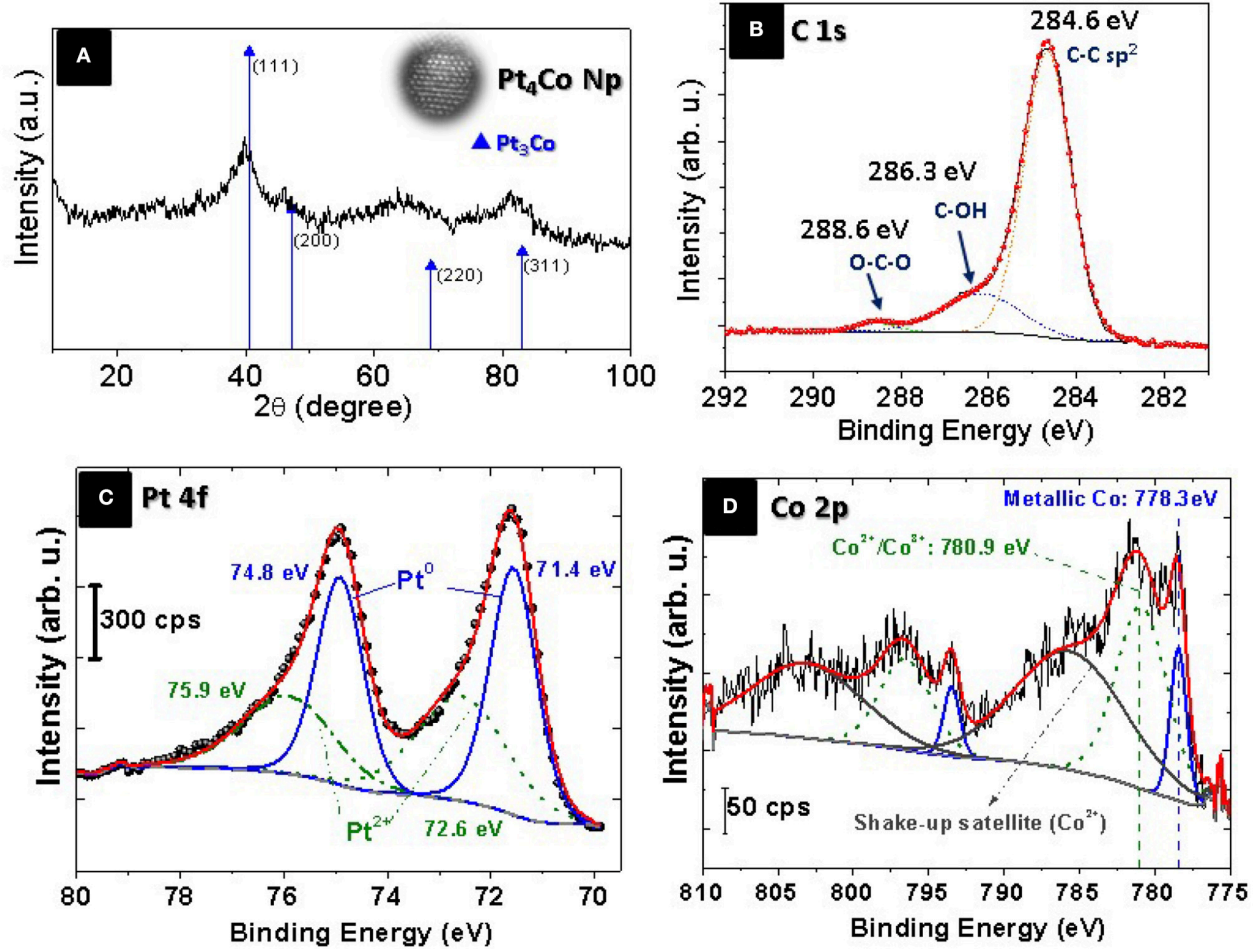
**(A)** XRD patterns of Pt_4_Co/C composite nanoparticles, and XPS spectra of: **(B)** C 1s region, **(C)** Pt 4f region and **(D)** Co 2p region showing the oxidation states of the Pt and Co in the Pt_4_Co/C composite nanoparticles.

By indexing X-ray diffraction (XRD) pattern (Figure 2A), we examined the crystalline structure of the Pt_4_Co/C composite nanoparticles. The XRD diffraction peaks are slightly shifted toward higher 2θ values compared to those of the cubic-face centered crystalline Pt (JCPDS card. No. 87-0646), peaks at 40.68°, 47.52°, 68.84°, and 82.36°, corresponding to the (111), (200), (220), and (311) planes, without any additional peak indicating that the Pt_4_Co/C catalyst maintains the face-centered cubic (fcc) structure of platinum. This shift can be due to the lattice contraction that occurs when larger Pt atoms are progressively substituted by smaller Co atoms, thus demonstrating the formation of bimetallic alloy nanoparticles (Xiong and Manthiram, 2004; Salgado et al., 2005; Huang et al., 2006; Jiang et al., 2009; Xia et al., 2015). Moreover, diffraction signals that could be associated to the presence of crystalline cobalt (JCPDS card. No. 01-1277) or its oxides have not been observed for the prepared binary Pt_4_Co/C composite nanoparticles. The cobalt contents within the Pt_4_Co/C sample derived from the Vergad's law (Santiago et al., 2007) was calculated by comparing the lattice parameter obtained from the measured (111) *2*θ Bragg peak (*a*_*exp*_ = 0.3850 nm) with those of the metallic Pt (*a* = 0.39231 nm; Swanson and Eleanor, 1953) and metallic cobalt (*a* = 0.35441 nm; Owen and Jones, 1954), respectively. The Co atomic content in the alloy, as determined by this XRD measurement, was about 19.3%. This is close to the nominal content 20%, detected by ICP and EDX analysis.

The oxidation states of Pt_4_Co/C catalyst were studied by X-ray photoemission spectroscopy (XPS). The survey XP-spectrum reveals the presence of carbon, cobalt, platinum and oxygen. The high-resolution C 1s spectrum is depicted in Figure 2B. The relative intensity of the peaks indicates primarily graphitic carbon C-C/C=C (284.6 eV) with small percentage of oxidized carbon as C-O and C=O species (286.3 and 288.6 eV), respectively (Arico et al., 1995). The analytical peaks related to Pt 4f orbitals and 2p orbitals for Co are shown in Figures 2C,D, respectively. The binding energies (BE) of 74.8 and 71.4 eV in Figure 2C correspond with Pt 4f_5/2_ and Pt 4f_7/2_, respectively, which can be splitted into two pairs of doublets: metallic Pt at 74.8 and 71.4 eV, while the second contribution at 75.9 and 72.6 eV correspond to Pt^2+^ species (Zsoldos and Guczi, 1992; Zheng et al., 2014a,b). The XPS signals in the Co 2p_3/2_ region (Figure 2D) reveal the presence of Co (0) at 778.5 eV shifted 0.4 eV toward higher BE in Pt-Co alloys compared with the monometallic cobalt phase at 778.1 eV) which is another confirmation of the formation of the Pt_3_Co alloy nanoparticle (Bardi et al., 1990). A Co 2p_3/2_ peak contribution at 780.9 eV can be assigned to both Co surface phase and Co oxide (Co^2+^/Co^3+^; Zsoldos and Guczi, 1992). Although, the Co 2p_3/2_ peaks are clearly accompanied by strong shake-up satellites, which are characteristic of CoO (Co^+2^), at 5 eV higher than its main peak, as well as a spin-orbit coupling of around 15.5 eV (Zsoldos and Guczi, 1992) the presence of Co_3_O_4_ (Co^3+^) cannot completely be ruled (Jimenez et al., 1995; Hueso et al., 2008). The low metallic Co content compared to the higher metallic Pt content confirms the oxide-cleansing action of Co addition (Arico et al., 2001). The electronegativity difference between Co and Pt (1.8 and 2.2, respectively; Shukla et al., 1999) implies an electron-drawing effect from Pt to the neighboring Co atoms, which make the latter more difficult to reduce.

To demonstrate the versatility and robustness of this synthesis method, we performed the one-step laser-driven pyrolysis of a toluene solution containing a mixture of both Pt(acac)_2_ and Co(acac)_3_ precursors in molar ratio of 1. The structure of the as-prepared Pt_x_Co_y_/C composite nanoparticles was characterized by TEM (Figure S3A) and STEM-HAADF (Figure S3C). The corresponding micrographs show highly crystalline spherical nanoparticles with average size of 2.3 ± 0.4 nm (Figure S3B) which are well-dispersed across the C matrix. The coexistence of Pt and Co elements in the particles is also demonstrated by the EDS analysis with the bimetallic composition of 55.5% and 44.5%, respectively (Figure S3D). The shift of XRD lines indicates proper alloying (Figure S4). Additional characterization by Raman and X-ray photoemission spectroscopy is provide in Figure S5. These results contributed to demonstrate that the proposed laser-assisted synthesis method is a general and feasible route for the synthesis of well dispersed, stable and highly pure C-encapsulated Pt_x_Co_y_ bimetallic nanoparticles. The nanoparticles were properly alloyed, while different stoichiometric ratios could be easily tuned by changing the the molar ratio of Pt and Co precursors.

### PROX catalytic tests

**Figure 3:**
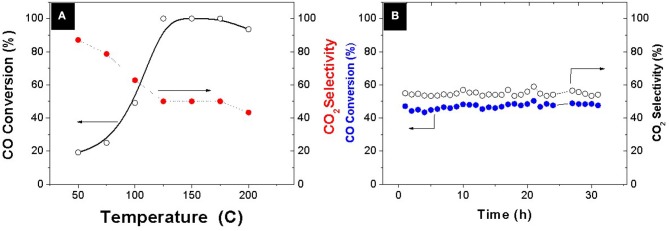
**(A)** Evolution of CO conversion and CO_2_ selectivity as a function of temperature **(B)** Time at T = 100°C. Feed composition: 1% CO, 1% O_2_, 3% H_2_O, 21% CO_2_, and H_2_ balance WHSW = 30 L·g^−1^·h^−1^.

A systematic evaluation of the catalytic capability of the Pt-Co alloy nanoparticles was performed after deployment onto an ETS-10 support. The preferential oxidation of CO in the presence of a hydrogen excess was thoroughly evaluated and compared with analogous bimetallic compositions available in the literature (Table 2). The evolution of CO conversion and selectivity as a function of temperature is presented in Figure 3A. The decrease in CO conversion at higher temperatures occurs due to oxygen scarcity caused by a surface fully covered with chemisorbed hydrogen and acceleration of undesirable concurrent hydrogen oxidation reaction which results in selectivity decrease. The carbon monoxide starts to convert at 50°C following a typical light-off curve and the total conversion is achieved at 125°C, which maintains constant in a temperature window of 50°C. The PROX unit is located between the outlet of the low temperature water gas shift reactor, working at 200°C, and the inlet of the fuel cell operating in the range of 80–120°C, in the case of low temperature proton exchange membrane fuel cell (PEMFC). Table 2 shows the operation temperature window reported by other authors, who synthesized bimetallic Pt-Co NPs by different methods. Except in the case of (Komatsu and Tamura, 2008), the highest temperature windows encountered in the literature, 40°C, correspond to the cases where the intermetallic Pt_3_Co phase has been identified by XRD or XRD and XPS. In our previous work (Lopez et al., 2016) we synthesized NPs by successive chemical impregnation at low temperature, where the intermetallic phase was discarded by XRD and XPS analysis. The bimetallic NPs consist of CoO_x_ on the surface of Pt, and the high activity at low temperature was associated to the presence of cobalt oxides that act as an oxygen reservoir. Oxygen is supplied by the reducible oxides to the CO adsorbed on the surface and the adsorption of oxygen on the surface, which is proposed as rate determining step in the case of non-promoted Pt catalysts, it is not necessary. However, the conversion in this work at a temperature as low as 50°C, is slightly higher, 20% compared to 12% (Lopez et al., 2016) at the same WHSV and similar metal load 1.4wt.%. Thus, the geometric effect associated to the Pt_3_Co alloy, widening the Pt-Pt atomic distance and electron transfer from Pt to Co, has lower sensitivity to temperature. The experimental results obtained in the present work remark the importance of the alloy formation in the catalytic results, increasing the activity of the catalyst, and widening the temperature operation window. Finally, the catalyst stability was tested for 30 h (Figure 3B) and no deactivation was observed.

**Table 2:** Comparison with literature data for bimetallic PtCo, including composition, preparation method, characterization of intermetallic phases and temperature operation window.

**References**	**Catalyst composition metal load % wt**.	**Preparation method**	**Active phases reported**	**WHSW (l g h^−1^)**	**Temperature operating window**
Yan et al., 2004	3%Pt 1%Co/γ-Al_2_O_3_	Sequential impregnation method Pt followed by Co. In both cases after impregnation calcination and reduction steps.	Pt_3_Co phase identified by XRD	40	120–160°C
Ko et al., 2007	0.5%Pt-Co/YSZ (Co/Pt = 5)	Sequential impregnation method Pt followed by Co. In both cases after impregnation calcination and reduction steps.	TEM: Isolated Pt-Co and Co nanoparticles. No XRD or XPS available	60	100–120°C
Snytnikov et al., 2007	2 % (Co–Pt)/C	Sequential impregnation of Pt and Co salts to obtain [Co(NH_3_)_5_NO_2_][Pt(NO_2_)_4_] 1.5H_2_O followed by reduction in H_2_	Co_0.5_Pt_0.5_ phase identified by XRD	N.A.	120–150°C
Komatsu and Tamura, 2008	Pt_3_Co/SiO_2_ (3% Pt and Pt/Co = 3)	Co-impregnation method	Pt_3_Co phase identified by XRD	8.4	180°C
Wang et al., 2013	Pt-Co/AlPO-5 (1%Pt-2%Co)	Co-impregnation method	EDX analysis of Pt-Co, presence of both metals	24	110–125°C
Furukawa et al., 2016	Pt_3_Co/MgO (3 wt%, Pt, Pt/Co = 3)	Co-impregnation method	Pt_3_Co phase identified by XRD	162	120–160°C
Lopez et al., 2016	1.4%PtCoOx/ETS-10	PtCoO_x_ NPs synthesized by sequential chemical wet reduction of Co and Pt salts, followed by incipient wetness impregnation of NPs in support	EDX analysis of Pt-Co, presence of both metals. Absence of Pt_3_Co phase discarded by XRD and XPS analysis	30	125–150°C
This work	1.4%Pt_4_Co/ETS-10	Pt_4_Co NPs synthesized by pyrolysis laser followed by incipient wetness impregnation of NPs in support	Pt_3_Co phase identified by XRD and XPS	30	125–175°C

Analogous wide temperature windows in PROX have been only reported when bimetallic alloy nanoparticles are present. Wang et al. (2013) prepared a PtCo catalyst (4%wt.) supported on AlPO_4_ and reported the complete removal of CO, in a simulated reformate stream at temperatures ranging from 80 to 130°C working at 30 L g^−1^ h^−1^ with an optimized catalyst containing a load of Pt as high as 4 and 0.7% Co supported on carbon nanotubes. Recently Furukawa et al. (2016) also reported a wide temperature window 90 to 160°C with a catalyst 3 wt.% Pt, Pt_3_Co supported in MgO at 162 L g^−1^ h^−1^ without the presence of CO_2_ and H_2_O.

## Conclusions

In summary, carbon-encapsulated alloy nanoparticles (Pt_x_Co_y_/C) have been successfully synthesized with controllable composition by a facile one-pot laser-assisted method. The carbon encapsulation strategy not only allows to prepare stable and well-dispersed bimetallic nanoparticles < 3 nm in size, but also prevents nanoparticles from agglomeration. The as-prepared Pt_4_Co/C catalyst has exhibited superior activity for the oxidation of CO in a simulated reformate gas stream. The solid containing 1.5 wt.% of nanoparticles can achieve complete CO conversion in a wide temperature window range of 125 to 175°C working at WHSV = 30 L h^−1^ g^−1^. The stability of this solid was tested for 30 h, showing no changes in CO conversion or selectivity. This work offers not only an important strategy to prepare stable Pt-Co bimetallic nanoparticles, but also provides an innovative alternative for the synthesis of high-performance catalysts, which may enable a wide variety of potentials applications in many fields, such as catalytic electrodes in the oxygen evolution reaction ORR.

## Author contributions

GM, AM, RM, and JS designed the experiments. GM, AM, and AL performed the reaction experiments. JLH contributed in the characterization. GM, AM, and AL designed and performed the experimental setup to determine the catalytic activity. GM, RM, and JS co-wrote the manuscript with the contribution and approval of all the authors.

### Conflict of interest statement

The authors declare that the research was conducted in the absence of any commercial or financial relationships that could be construed as a potential conflict of interest.
