# Supplementary material for: Laser-Assisted Production of Carbon-Encapsulated Pt-Co Alloy Nanoparticles for Preferential Oxidation of Carbon Monoxide
Source: Front Chem. 2018 Oct 16;6:487. doi: 10.3389/fchem.2018.00487 (PMC6198107; doi:10.3389/fchem.2018.00487)
Supplement: Supplementary file 1 [file Data_Sheet_1.pdf]

Supplementary Material

Laser-assisted production of carbon-encapsulated Pt-Co alloy nanoparticles for preferential oxidation of carbon monoxide

G Martinez[1,2]*, A Malumbres[2], A Lopez[2], R Mallada[1,2], J L Hueso[1,2] and J Santamaria[1,2]*

*** Correspondence:** Jesus Santamaria: jesus.santamaria@unizar.es and Gema Martinez: gemamar@unizar.es

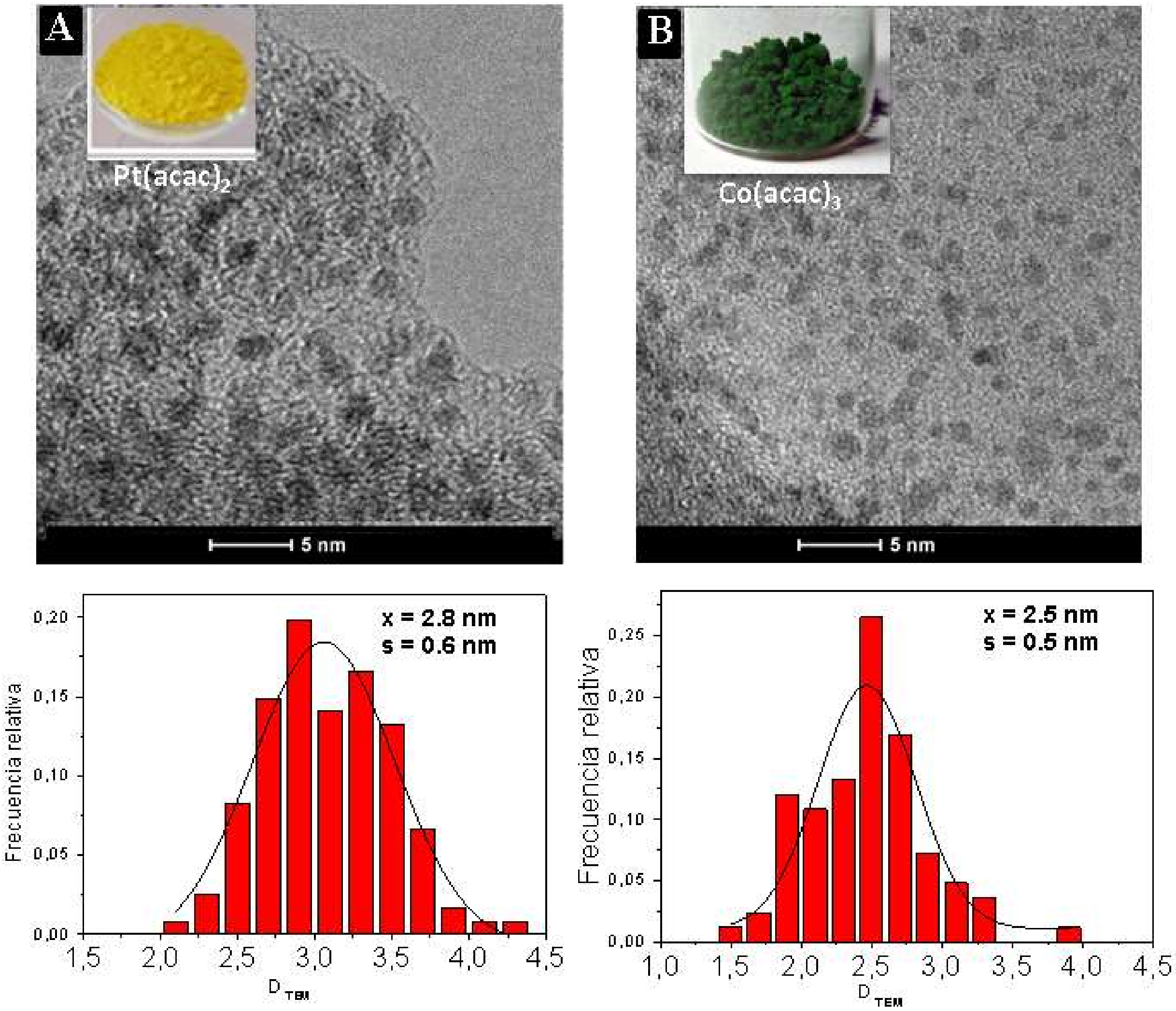

Supplementary FIGURE 1. TEM micrographs and particle size distributions corresponding to monometallic nanoparticles obtained after laser pyrolysis of one precursor: (A) platinum oxide nanoparticles (PtO/C) and (B) cobalt oxide nanoparticles (Co_2O_3/C) embedded into a carbon matrix.

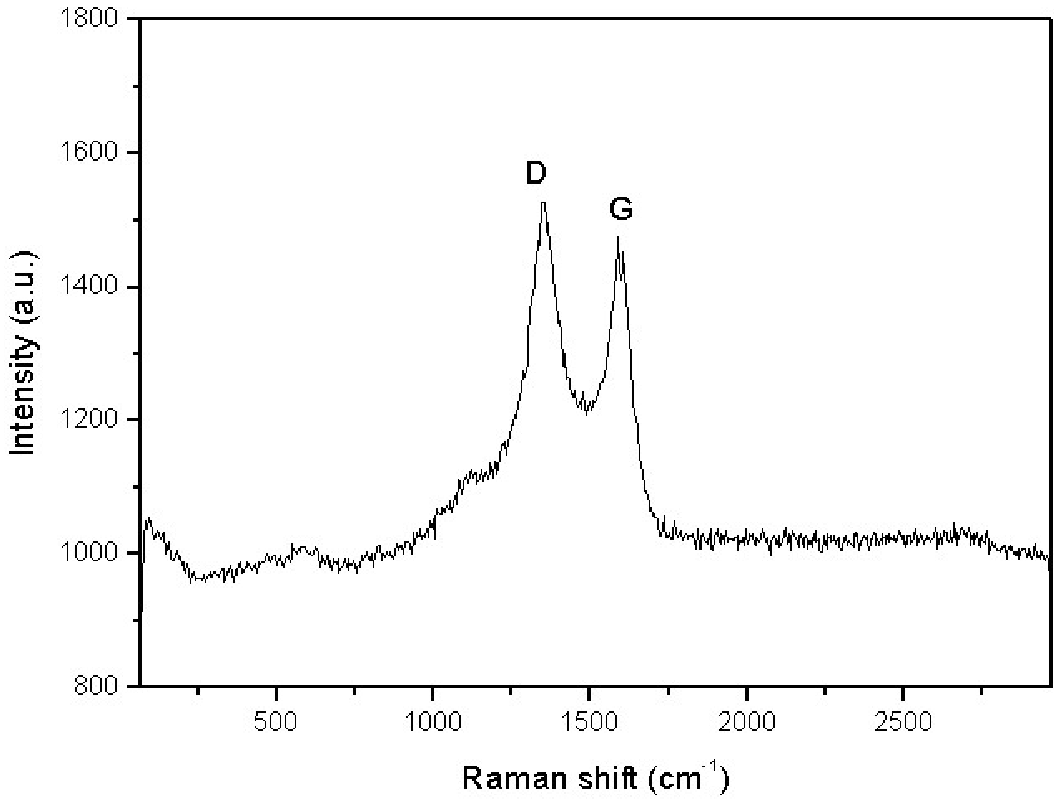

Supplementary FIGURE 2. Raman spectrum of the Pt_4Co/C composite nanoparticles.

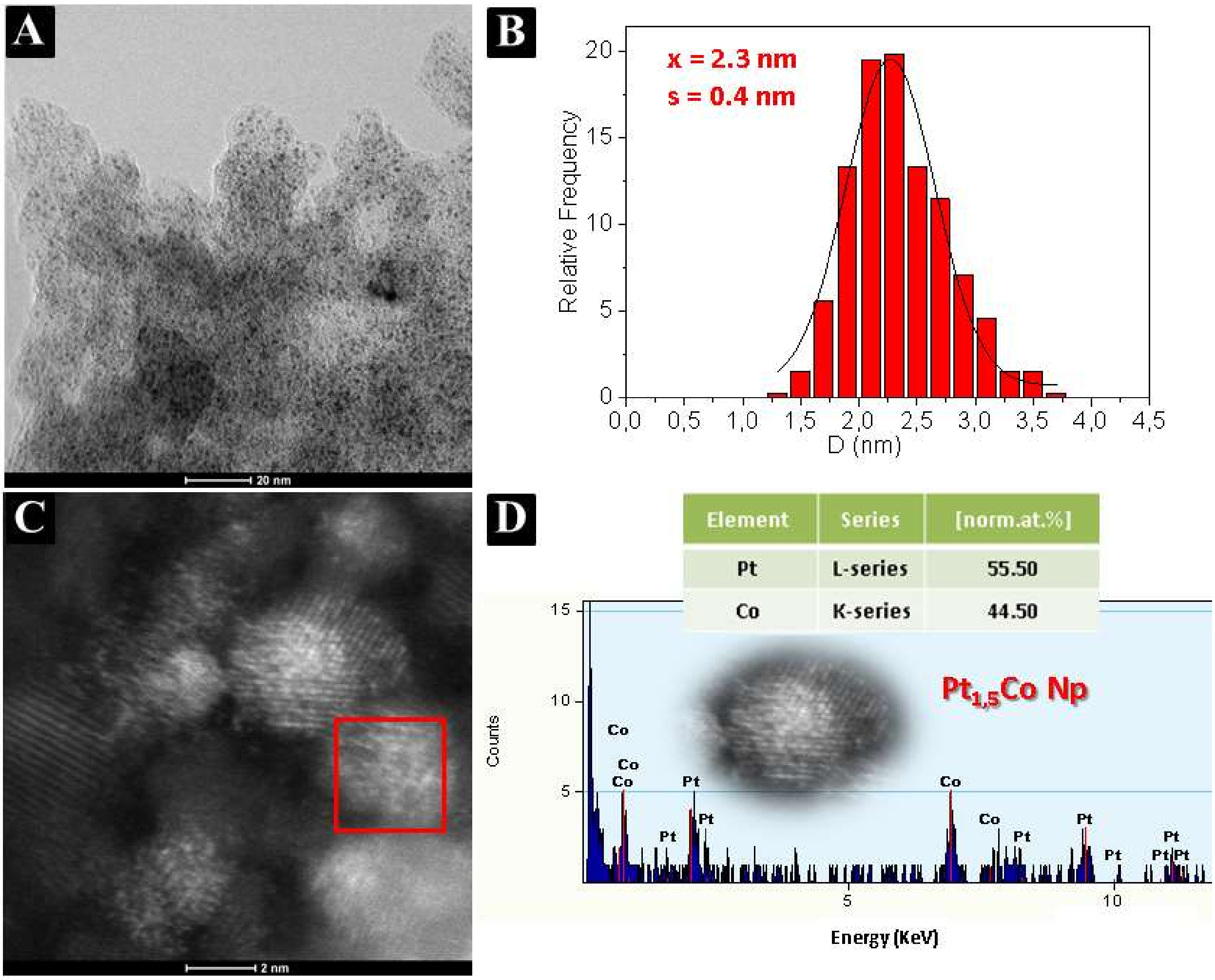

Supplementary FIGURE 3. Characterization of the Pt-Co/C nanoparticles synthesized when the molar ratio of starting Pt:Co precursors is set to 1: (A) Low magnification TEM micrograph, (B) particle size histogram, (C) STEM-HAADF micrograph acknowledging the crystalline order and the presence of different atomic contrast and (D) EDS analysis of an individual particle (selected area in Figure S3 C) indicating an experimental atomic ratio of Pt to Co of approximately 1.5 ($Pt_{1.5}Co/C$).

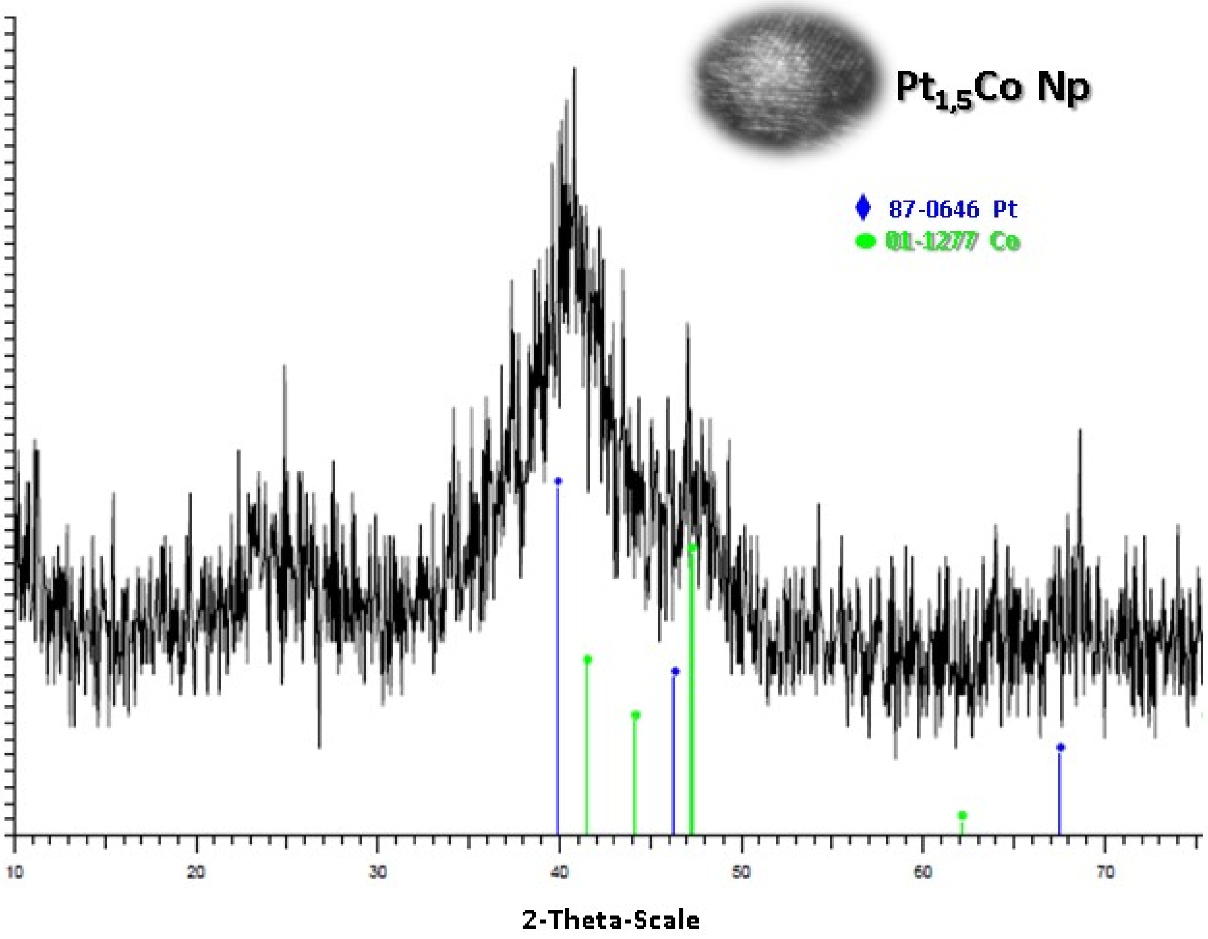

Supplementary FIGURE 4. XRD patterns of $Pt_{1.5}Co/C$ composite nanoparticles including the reference patterns for Pt and Co.

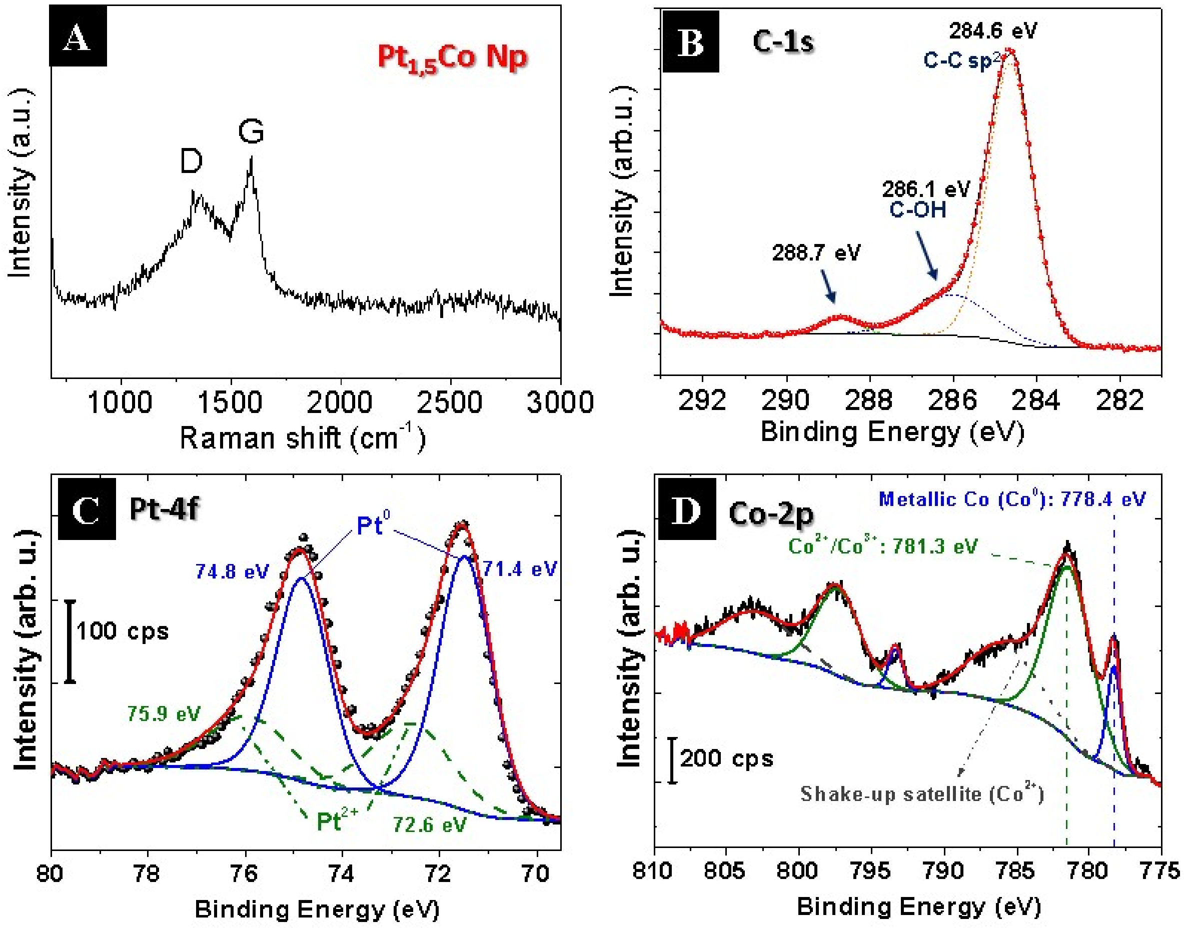

Supplementary FIGURE 5. (A) Raman spectrum of the $Pt_{1.5}Co/C$ composite nanoparticles and XPS spectra of: (B) C 1s region, (C) Pt 4f region and (D) Co 2p region showing the oxidation states of the Pt and Co in the $Pt_{1.5}Co/C$ composite nanoparticles.
